# DArTseq-Based, High-Throughput Identification of Novel Molecular Markers for the Detection of Blackleg (*Leptosphaeria* Spp.) Resistance in Rapeseed

**DOI:** 10.3390/ijms25158415

**Published:** 2024-08-01

**Authors:** Ewa Starosta, Tomasz Jamruszka, Justyna Szwarc, Jan Bocianowski, Małgorzata Jędryczka, Magdalena Grynia, Janetta Niemann

**Affiliations:** 1 Department of Genetics and Plant Breeding, Poznań University of Life Sciences, Dojazd 11, 60-632 Poznań, Poland; ewa.starosta@up.poznan.pl (E.S.); tomasz.jamruszka@up.poznan.pl (T.J.); justyna.szwarc@up.poznan.pl (J.S.); 2 Department of Mathematical and Statistical Methods, Poznań University of Life Sciences, Wojska Polskiego 28, 60-627 Poznań, Poland; jan.bocianowski@up.poznan.pl; 3 Institute of Plant Genetics of the Polish Academy of Sciences, Strzeszyńska 34, 60-479 Poznań, Poland; mjed@igr.poznan.pl; 4 IHAR Group, Borowo Department, Strzelce Plant Breeding Ltd., Borowo 35, 64-020 Czempiń, Poland; m_grynia@hr-strzelce.pl

**Keywords:** blackleg resistance, *Leptosphaeria* spp., *Leptosphaeria maculans*, *Leptosphaeria biglobosa*, molecular markers, GWAS, SNP, DArTseq, next-generation sequencing

## Abstract

Blackleg disease, caused by *Leptosphaeria* spp. fungi, is one of the most important diseases of *Brassica napus*, responsible for severe yield losses worldwide. Blackleg resistance is controlled by major *R* genes and minor quantitative trait loci (QTL). Due to the high adaptation ability of the pathogen, *R*-mediated resistance can be easily broken, while the resistance mediated via QTL is believed to be more durable. Thus, the identification of novel molecular markers linked to blackleg resistance for *B. napus* breeding programs is essential. In this study, 183 doubled haploid (DH) rapeseed lines were assessed in field conditions for resistance to *Leptosphaeria* spp. Subsequently, DArTseq-based Genome-Wide Association Study (GWAS) was performed to identify molecular markers linked to blackleg resistance. A total of 133,764 markers (96,121 SilicoDArT and 37,643 SNP) were obtained. Finally, nine SilicoDArT and six SNP molecular markers were associated with plant resistance to *Leptosphaeria* spp. at the highest significance level, *p* < 0.001. Importantly, eleven of these fifteen markers were found within ten genes located on chromosomes A06, A07, A08, C02, C03, C06 and C08. Given the immune-related functions of the orthologues of these genes in *Arabidopsis thaliana*, the identified markers hold great promise for application in rapeseed breeding programs.

## 1. Introduction

*Brassica napus* (genome AACC, 2n = 38) is one of the most widespread oilseed crops in the world [1], known as rapeseed or canola. Rapeseed is an amphidiploid species that originated from two closely related species: *Brassica rapa* (AA, 2n = 20) and *Brassica oleracea* (CC, 2n = 18) as a result of interspecific hybridization and chromosome doubling. Its formation is dated to about 7500 years ago [2]. Until the 1970s, rapeseed oil was not considered suitable for consumption, due to its less than favorable taste and high erucic acid (EA) and glucosinolates (GSLs) content [3]. EA is considered to be toxic to humans and animals. Several studies performed on rats stated the presence of cardiotoxic properties due to the incomplete metabolism of EA in the body, resulting in the accumulation of triacylglycerols [4]. Furthermore, GSLs are known for their antimicrobial and anticarcinogenic properties; however, they can turn into anti-nutritious agents. Moreover, the bitter taste of some of the GSLs affect the palatability of the seed oil [5]. Rapeseed oil became suitable for consumption with the deployment of “double-zero” or “00” cultivars with the content of EA <2% and the meal having less than <25 mmol/kg at a moisture content of 9% of glucosinolates [6,7]. Currently, the term “canola” refers to edible cultivars of rapeseed with a lowered content of erucic acid and glucosinolates (“00”) and is a trademark for the Canadian Canola Association [8]. Rapeseed is one of the major players in the global oilseed market, ranking third in terms of oil production, right behind soybean and palm. In Poland, its production area reached 1.1 Mha in 2023 with a yield of 3.32 t/ha (stat.gov.pl). Rapeseed is a relatively demanding crop, as its yield depends on both biotic and abiotic factors such as soil quality, environmental conditions and the occurrence of diseases [8,9]. Regrettably, the reliance on monoculture cultivation for rapeseed creates favorable conditions for the uncontrolled spread of pathogens.

Blackleg is one of the most economically important diseases affecting *Brassica* crops. Its casual agents are *Leptosphaeria maculans* and *Leptosphaeria biglobosa* (new classification: *Plenodomus lingam* and *Plenodomus biglobosus*, respectively). This disease causes significant losses worldwide, exceeding 10% annually. In some cases, reports indicate losses as high as 50% [10,11]. Yearly loss in Poland reaches 10–40%, depending on the region [12]. Thus, *L. maculans* is a major threat for global rapeseed production. It is a hemibiotrophic fungi that can infect the host in every stage of plant development. However, the most severe plant damage is observed between the germination of seeds and the sixth leaf growth stage [13]. The initial symptom of blackleg is the appearance of gray lesions scattered on cotyledons and lower leaves with black pycnidia. As the infection progresses, basal stem cankers emerge and the lesions spread to the upper leaves, stems and siliques [7]. The high adaptability of the pathogen poses another challenge to rapeseed production. Its ability to reproduce both sexually (ascospores) and asexually (pycnidiospores) is the primary driving force of the pathogen evolution. Furthermore, *L. maculans* can survive in dry and cold environments as a saprophyte in crop residues. The propagation of the fungi occurs over both short and long distances through wind dispersal [14,15].

Several approaches to blackleg disease control have been utilized. Soil tillage, stubble management, crop rotation, the adjustment of sowing date, the use of microbiological control agents and fungicide application are only a few examples of methods used to limit *L. maculans* infections [16,17,18]. However, one of the most sustainable strategies is the use of resistant cultivars of canola. Several rapeseed varieties with medium to high blackleg tolerance have been released and commercialized. They utilize both race-specific, major gene (*R* gene)-mediated resistance and non-race-specific, polygenic resistance (quantitative trait loci, QTL). To date, at least 20 blackleg *R* genes have been identified, but only five of them have been cloned: *Rlm2*, *Rlm4*, *Rlm7*, *Rlm9* and *LepR3* [19,20,21,22]. Single or pyramided *R* genes are commonly used in commercial cultivars of rapeseed, such as Aganos (*Rlm7*, Syngenta) and LG Scorpion (*Rlm7*, Limagrain), as well as Surpass 400 (*Rlm1*, *RlmS*), Jet Neuf (*Rlm4*) and Skipton (*Rlm4, Rlm9*), which are used as differential lines in host–pathogen interaction research [23,24]. However, their effectiveness against pathogens can diminish over time due to the evolution of new pathogen strains. Polygenic resistance is considered to be more durable and, when combined with race-specific resistance, may provide long-term protection [25,26,27].

The development of new resistant canola cultivars through traditional methods that rely mainly on the selection of genotypes with a favorable phenotype is time- and work-consuming. Currently, Marker Assisted Selection (MAS) is becoming one of the main methods of selection for resistant germplasm. Thus, its practical usefulness in resistance breeding is undeniable. However, due to the large complexity of the *B. napus* genome, identifying the exact location of resistance genes is challenging. This hinders the establishment of linked molecular markers [28,29]. Fortunately, with the increasing availability of many publicly accessible rapeseed reference genomes and gene assemblies, MAS becomes easier and is now one of the main research trends in *B. napus* breeding. Additionally, most studies focusing on the identification of novel molecular markers employ the use of doubled haploid (DH) lines–fully homozygous breeding lines. Their greatest advantage is the reduced breeding time in comparison to conventional methods of backcrossing and selfing. Rapeseed DH production is an extensively investigated topic [30]. Closely linked molecular markers can support and accelerate the development of new rapeseed varieties with high resistance levels. Combined with DH lines, the breeding time can be considerably shortened in both the marker development phase as well as in the selection of homozygous genotypes.

Diversity Arrays Technology sequencing (DArTseq^TM^) is a method that provides high-throughput data in a relatively short timeframe. It utilizes a reduction in genome complexity and Next-Generation Sequencing (NGS) to generate large numbers of two types of molecular markers: SNP (Single Nucleotide Polymorphism) markers and SilicoDArT markers. It has been successfully applied in genetic studies of numerous crops and genotypes [31,32,33,34].

This study aims to identify new molecular markers linked to blackleg resistance in rapeseed DH lines using the means of NGS and physical mapping.

## 2. Results

### 2.1. Phenotyping

**Figure 1:**
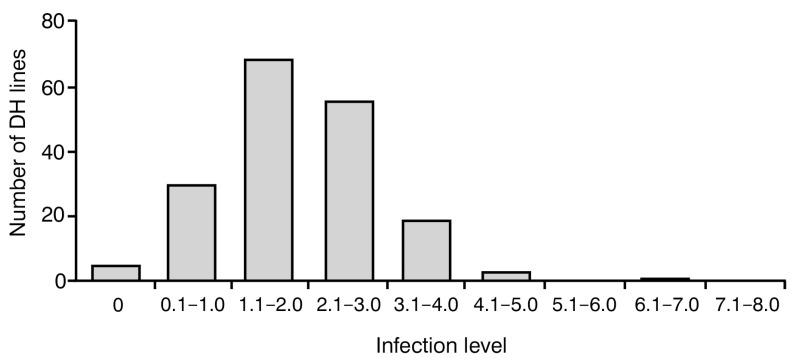
Frequency distribution of blackleg infection levels in 183 analyzed *Brassica napus* doubled haploid (DH) lines. Score 0 indicates no visible damage; score 9 indicates completely damaged plant.

To investigate the level of resistance to blackleg in analyzed 183 DH lines, the frequency of disease symptoms was assessed using a 0–9 severity scale. On this scale, 0 indicates no visible disease damage and 9 indicates a completely damaged plant. The analysis of variance showed a significant effect (*p* < 0.001) in their genotype on the observed degree of resistance to blackleg. Notably, all plants were infected with *Leptosphaeria* spp. in varying degrees, ranging between 0 and 7, with an average value of 2.05 ± 1.06. Most of them (179) displayed satisfactory blackleg disease resistance with a score of 0–4. No plants with infection levels of 8–9 were observed (Figure 1).

### 2.2. Genotyping

**Figure 2:**
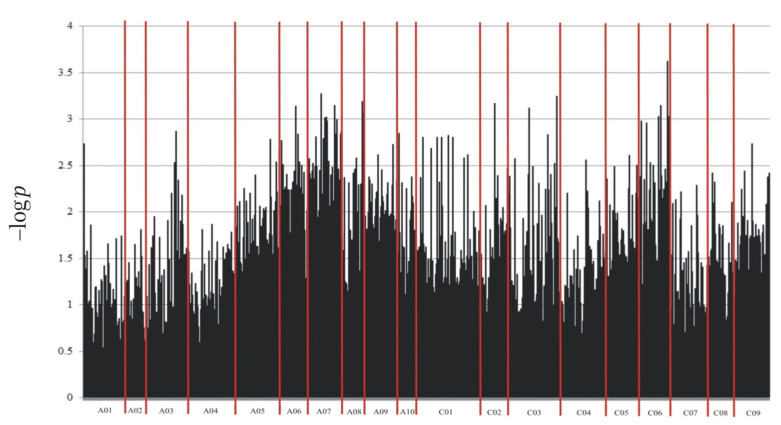
Manhattan plot for polymorphic markers associated with resistance of *B. napus* to blackleg.

**Figure 3:**
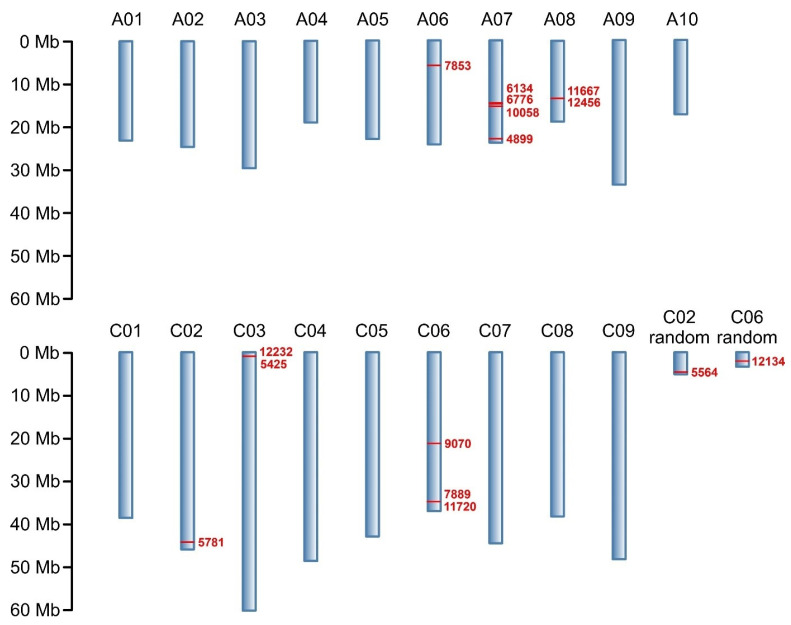
Physical map of markers associated with resistance of *B. napus* to blackleg at *p* < 0.001. Chromosomes are shown in blue; chromosome numbers are annotated above. Reported markers and their positions on chromosomes are shown in red. Marker locations are expressed in megabases (Mb) as indicated by the scale.

**Figure 4:**
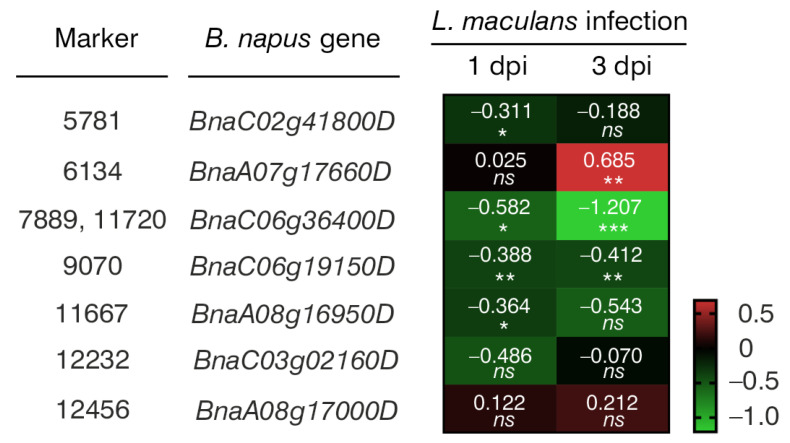
A heatmap showing changes in the expression levels of seven *B. napus* genes that harbor selected markers caused by *Leptosphaeria maculans* 1 and 3 days post-inoculation (dpi). The heatmap depicts log2-fold changes in transcriptomic data acquired from Becker et al. (2019). Intensity of red indicates upregulated gene expression, while the intensity of green indicates downregulated gene expression. Significant differences from the mock-treated plants were determined using two-tailed Student’s *t*-test. * *p* < 0.05; ** *p* < 0.01; *** *p* < 0.001; *ns*, not significant.

Through NGS, a total of 133,764 molecular markers (96,121 SilicoDArT and 37,643 SNP) were obtained. The significance of the identified markers was determined using MAF >0.25 and the number of missing observations <10%. Following this criteria, 12,948 (10,008 SilicoDArTs and 2940 SNPs) markers were selected for association mapping using GWAS (Figure 2). Importantly, fifteen (nine SilicoDArT and six SNP) markers from the latter group were associated with plant resistance to *Leptosphaeria* spp. at the highest significance level, *p* < 0.001 (Table 1). The highest number of them (4 and 3, respectively) was present on A07 and C06 chromosomes (Figure 3). Further analyses also revealed that eleven of these fifteen markers were located within only ten genes, due to the presence of the 7889SilicoDArT and 11720SNP marker in the same gene, *BnaC06g36400D* (Table 2). The potential role of these genes as candidate genes for rapeseed defense against *L. maculans* was assessed with a use of transcriptomic data from Becker et al. (2019) [34]. Out of seven genes found in the latter work, five exhibited significant changes in expression within three days after the pathogen inoculation, namely *BnaC02g41800D*, *BnaA07g17660D*, *BnaC06g36400D*, *BnaC06g19150D* and *BnaA08g16950D* (Figure 4).

## 3. Materials and Methods

### 3.1. Plant Material

The plant material consisted of 183 DH lines of rapeseed (*B. napus* L.) derived from Strzelce Plant Breeding Ltd. (Strzelce, Poland) IHAR Group in Borowo. The DH lines were obtained by performing intraspecific crosses of selected registered rapeseed varieties with known blackleg resistance in order to represent a diverse disease response structure in the mapping population.

### 3.2. Field Assessment

All DH lines were assessed in terms of the resistance to blackleg in field conditions. The trials were conducted on Strzelce Plant Breeding Ltd. IHAR Group Borowo test fields. The experiment was set up in a completely randomized block design and included three replications. The observations were completed on adult plants in the BBCH 70–89 phase. The level of blackleg resistance was determined using disease symptoms evaluation according to the 0–9 scale elaborated by Jedryczka (2006) [35], in which score 0 means no visible disease symptoms and score 9 means a completely damaged plant with numerous pycnidia on the stem and leaf surfaces. The details of the evaluation scale were as follows:

0 healthy plant with no visible disease symptoms;
1 slight surface discoloration of the main stem;
2 discoloration covering up to 10% of the main stem;
3 discoloration covering from 11% to 20% of the main stem;
4 discoloration covering from 21% to 30% of the main stem, a few pycnidia;
5 discoloration covering from 31% to 50% of the main stem, several pycnidia;
6 discoloration covering from 51% to 75% of the main stem, numerous pycnidia;
7 discoloration covering over 76% of the main stem and parts of side branches, numerous pycnidia;
8 discoloration covering the whole main stem and extending to side branches, numerous pycnidia, smaller number of siliques as compared to the healthy plants;
9 dead plant with thin stem, very small number or no siliques, numerous pycnidia on the main stem and side branches.

Like in all visual assessments, this scale was an indication for the evaluator, but variations were possible.

### 3.3. DNA Extraction

Whole genomic DNA was extracted from young seedlings using a Genomic Mini AX Plant kit (A&A Biotechnology, Gdańsk, Poland) according to supplied protocol. The concentration and purity of the isolated DNA was determined using the spectrophotometer DeNovix DS-11, DeNovix, Wilmington, DE, USA. Samples with a DNA concentration higher than 100 µL and 260/280, 260/230 ratios of ~1.8 and ~2.0, respectively, were diluted with Tris-HCl buffer to 100 ng/µL in 25 µL volume.

### 3.4. Genotyping

The genomic DNA isolated from 183 DH lines, previously assessed in field conditions, was sent to Diversity Arrays Technology (University of Canberra, Australia) for DArTseq analysis. In the latter procedure, whole genomic material was digested by selected restriction enzymes to reduce the complexity of the genome [36]. This approach allowed the fragmentation of DNA samples in a highly reproducible manner and the consequent selection of the predominantly active, low-copy sequence areas, which are the most informative for genetic analysis. Next, the digested fragments underwent ligation with adaptors and amplification using Polymerase Chain Reaction (PCR). Equimolar quantities of amplification products from each genotype were combined to create gene pool representation, followed by sequencing with Illumina Hiseq2000. Generated reads were processed with DArT analytical pipelines (Diversity Arrays Technology, Australia) to filter poor quality sequences and to identify sequences for marker calling. Only markers meeting the following criteria were selected for the association analysis: one marker within a given sequence (69 nt), an allele frequency (MAF) >0.25 and a missing observations fraction <10%.

The physical map positions of identified markers were determined against the Darmor-bzh v4.1 reference genome. Obtained metadata (i.e., presence/absence and localization of identified SilicoDArTs and SNP markers) were further subjected to statistical analysis.

The distances between the markers and the nearest genes were measured using the JBrowse tool against the Darmor-bzh 4.1 assembly, available through the *B. napus* multi-omics information resource (BnIR, https://yanglab.hzau.edu.cn/BnIR (accessed on 22 March 2024)) platform [37].

### 3.5. Statistical Analysis

Data were further subjected to analysis of variance in a model with random effects of genotypes. Association mapping with Genome-Wide Association Studies (GWAS), based on the SilicoDArT and SNP data and the average resistance to blackleg values, was conducted using the method based on the mixed linear model with a population structure estimated via eigenanalysis and modeled using random effects [38,39]. All analyses and visualizations of the results were performed in GenStat 23.1 [40], using the QSASSOCIATION procedure. QSASSOCIATION performs a mixed model marker—trait association analysis (also known as linkage disequilibrium mapping) with data from a single-environment trial. To avoid false positives in association mapping studies, a control is necessary for genetic relatedness. The model used was specified using the RELATIONSHIPMODEL = eigenanalysis option, which infers the underlying genetic substructure in the population by retaining the most significant principal components from the molecular marker matrix [41]; the scores of the significant axes are used as covariables in the mixed model, which effectively is an approximation to the structuring of the genetic variance covariance matrix by a coefficient of the coancestry matrix (kinship matrix) [31]. The significance of association between the resistance to blackleg and SilicoDArT and SNP markers was assessed using *p* values corrected for multiple testing using the Benjamini–Hochberg method.

### 3.6. Functional Annotation of Genes

The functional annotation of the potential involvement of selected genes in *B. napus* defense mechanisms against *L. maculans* was determined based on transcriptomic data from Becker et al. (2019) [42], available on the BrassicaEDB platform (https://brassica.biodb.org/ (accessed on 25 March 2024)).

## 4. Discussion

The *Leptosphaeria* spp. fungal complex is a great threat to rapeseed crop cultivation worldwide [43], especially after warm and wet autumns [44]. The breakdown of resistance in commercial rapeseed cultivars emphasizes the importance of searching for novel resistance sources in rapeseed breeding. Molecular markers are becoming an essential tool for the selection of resistant plants. Currently, the trends in modern farming and Integrated Pest Management programs require innovative and environmentally friendly solutions, allowing effective protection against diseases and pests [45,46,47]. Plant resistance breeding combined with MAS is one of such solutions. Developing molecular markers linked with blackleg resistance is an extremely complex and tremendous task.

The recently observed reduction in the cost of sequencing platforms has increased the efficiency of GWAS. Moreover, the ability of obtaining a large number of markers in relatively short time makes GWAS an incredibly useful tool for association mapping and for uncovering the genetic architecture of agronomically important phenotype characteristics.

The GWAS technique was successfully used in numerous *Brassica* studies to discover genome regions associated with both quality and resistance traits. For instance, GWAS has been used in the identification of SNP markers connected to glucosinolate and hemicellulose seed content [48], the identification of flowering-related traits [49] and for associating SNPs with resistance to *Plasmodiophora brassicae* [50]. Furthermore, the combination of GWAS, QTL mapping and expression analysis led to the recognition of a candidate gene controlling the branch number in rapeseed [51]. For blackleg resistance, several markers connected to major *R* genes and QTLs in Brassicas were also discovered, e.g., candidate *Rlm6* gene in *B. juncea* [52], QTL resistance loci in *B. napus* [53,54] and candidate *LepR1* genes in *B. napus* [55]. The recent discoveries deepened the knowledge of the genetic architecture of the blackleg resistance and offered new perspectives on complex pathogen–host relationships.

As previously mentioned, the *L. maculans*–*B. napus* interaction employs two types of resistance. The first one, qualitative resistance mediated by major *R* genes, is relatively well recognized and studied. In this type, it is believed that the *L. maculans*–*B. napus* pathosystem follows Flor’s gene-for-gene theory [56]. Thus, the successful deployment of resistant cultivars and the control of blackleg disease relies on the recognition of host and pathogen genotype. The characterization of single *R* genes is possible through phenotypic tests that use inoculation [57]. However, such tests are often very laborious, time consuming and require an extensive collection of precisely described pathogen isolates. The use of molecular markers enables the exact characterization of deployed varieties and the establishment of a rotation strategy in a shorter and more convenient way [58]. On the other hand, the second type of resistance, quantitative resistance (QTL), is poorly understood. It is mediated by several minor genes with small effect. Additionally, QTLs have low heritability and a non-Mendelian and accumulative character. It is challenging to pinpoint the exact genes or molecular markers linked with qualitative blackleg resistance because of the strong genotype x environment effect. Moreover, the method of evaluation, localization and the selection of winter, semi-winter and spring type rapeseed may affect the outcome of the analysis [59]. To date, multiple QTLs have been located on all 19 chromosomes of *B. napus*, but few have proved to be effective in various environmental conditions [60,61,62,63].

In this work, we evaluated the resistance of 183 rapeseed DH lines to blackleg via the observation of disease symptoms in adult plants (Figure 1). The analysis of variance showed a significant effect in their genotype on the observed degree of resistance to blackleg.

Among 12,948 markers selected for association mapping, 15 markers located on the *B. napus* chromosomes A06, A07, A08, C02, C03, C06 and C08 were found to be linked to blackleg resistance at the highest significance level, *p* < 0.001. Their greatest concentration was observed on chromosome A07 (Figure 3). This finding aligns with previous estimations indicating that this chromosome harbors the most *R* genes, which are responsible for disease resistance in plants [64]. However, it is crucial to emphasize that the A07 chromosome is not the sole repository of *R* genes against fungal infections. Rather, this group is dispersed throughout the rapeseed genome, as evidenced by the presence of the selected markers on several other chromosomes.

Out of these 15 markers, 11 were found within 10 *B. napus genes* (*BnaC02g41800D*, *BnaA07g17660D*, *BnaA07g18190D*, *BnaA06g11460D*, *BnaC06g36400D*, *BnaC06g19150D*, *BnaA07g19340D*, *BnaA08g16950D*, *BnaC03g02160D* and *BnaA08g17000D*) (Table 2). While SilicoDArT marker sequences were more evenly distributed between introns and exons (three exonic, one intronic and two across both these gene elements), most of the identified SNPs (four of all five) were located in exons (Table 2). This discrepancy can be related to the more complex structure of SilicoDArT markers that often contain indels and SNPs, sometimes in combination, and span a larger genomic region than single SNPs (https://www.diversityarrays.com/ (accessed on 17 November 2023)). The SNP in marker 100058 was responsible for the occurrence of alanine (GCT in gene sequence) in relation to the valine (GTT) in the non-marker sequence that makes a missense mutation. The SNP in marker 12232 also contributes to a missense change encoding for serine (TCT) as an alternative to threonine (ACT). A change in SNP in marker 11720 to a non-marker sequence causes a nonsense mutation with a switch of cysteine-encoding TGT to a STOP codon. Thereby, these three SNPs can have a direct impact on the length and properties of the encoded proteins. There was also found a synonymic mutation where the ATC codon for isoleucine in the 11667 SNP marker is changed to another codon for this amino acid, ATT. In light of recent reports, despite the absence of an amino acid change in the protein, such a mutation may also not be neutral [65]. This would explain the observed linkage of the 11667 marker with rapeseed resistance to fungal pathogens.

Interestingly, the expression of half of these 10 genes showed statistically significant changes following *L. maculans* inoculation (Figure 4), suggesting their potential involvement in rapeseed’s defense against fungal pathogens. Similarly, according to the literature review, several of their orthologues in *Arabidopsis thaliana* were verified to take part in plant immune response to pathogen infection (Table 3). For example, *A. thaliana At5g08130* (orthologue of *BnaC02g41800D*) encodes a basic helix–loop–helix transcription factor that is involved in brassinosteroid signaling upon stress conditions. Brassinosteroids appear to modulate plant interactions with pathogens. Their effect is dependent on plant and pathogen species [66]. In transgenic *B. napus* plants, the overexpression of the brassinosteroid biosynthetic gene AtDWF4 results in increased tolerance to environmental stresses, including a resistance to *L. maculans* and *Sclerotinia sclerotiorum* [67,68] (Table 3).

*At1g16980* (orthologue of *BnaA06g11460D*) encodes trehalose-6-phosphate synthase. The involvement of several trehalose-6-phosphate synthases and trehalose in increasing plant resistance to pathogens has been proved a number of times [69,70,71,72] (Table 3). Shi et al. (2019) suggested that trehalose may trigger signal transduction pathways [73].

*At3g62600* (orthologue of *BnaA07g19340D*) encodes the DnaJ heat shock protein (HSP40) family member that has been extensively studied both in plants and animals. It takes part in protein folding and is involved in regulatory pathways of many physiological processes. The latter confer salt stress tolerance, drought tolerance, temperature stress and development [74,75,76,77] (Table 3). Liu et al. (2022) found that 16 out of 17 *TaDnaJs* were upregulated after wheat yellow mosaic virus infection, suggesting a role for DnaJs in plant defense responses [78]. It was also observed that the overexpression of the chloroplast-targeted *DnaJ* gene, *LeCDJ2*, confers a higher resistance to *Pseudomonas solanacearum* in tomato plants [79]. Moreover, Zhong et al. (2017) established that the silencing of OsDjA6 results in a higher resistance of rice to rice blast caused by *Magnoporthe oryzae* (*Pyriculoria oryzae*). It was concluded that this protein negatively regulates rice immunity to the pathogen [80].

*At4g38690* (orthologue of *BnaA08g16950D*) encodes the phospholipase C-like (PLC) type of proteins that are commonly associated with plant immune pathways (Table 3). They are multidomain proteins with enzyme activity, involved in phosphoinositide metabolism and calcium signaling. Phosphoinositide-specific phospholipase C (PI-PLC) enzymes act downstream of the recognition of pathogens via immune receptors [81]. It was found that PLCs are crucial for hypersensitive (HR) immune response in plants. HR is attributed to resistance response in rapeseed mediated by major *R* genes [55]. The effectors of several plant pathogens such as *Cladosporum fulvum*, *Pseudomonas syringae* and *Xanthomonas oryzae* induced the accumulation of phosphatidic acid (PA). The activation of PI-PLC leads to the formation of PA as the final product [82,83]. PA is considered to be a messenger in the immune response pathway [84,85]. Due to high homogenicity of *B. napus* and *A. thaliana* genes, functional information can be considered as consolidated across the two species.

In addition to the chromosomes reported in this work, similar GWAS studies also found SNP and DArT markers related to blackleg resistance in other *B. napus* genomic localizations. The selected markers were linked to both *R* genes and QTLs [54,60,86,87]. Raman et al. (2016) identified 36 molecular markers located on 13 *B. napus* chromosomes. One major QTL observed on chromosome A01 explained as much as 14.7% of the phenotypic variation. Another study reported the identification of *Rlm12* loci on chromosome A01 [86]. Most recent studies combine GWAS and Whole Genome Sequencing (WGS) methods in multi-trait, multi-year analyses. While most of localized regions are linked to known *Rlm* regions, tens of new potential resistance regions are detected. Several of these regions hold putative genes with functions related to stress response and disease defense [54,63,87,88].

In conclusion, this study employed a high-throughput approach to identify fifteen molecular markers (SilicoDArT and SNP type) associated with *B. napus* resistance to blackleg at the significance level of *p* < 0.001. Eleven of them were localized within ten genes. To evaluate the potential involvement of these genes in *B. napus* immune responses against fungal pathogens, a preliminary characterization was conducted based on either available *B. napus* transcriptomic data or known functions of *A. thaliana* orthologues described in the literature. All of them were found to take part in stress as well as immune response pathways to pathogen infection. This suggests that the identified molecular markers are indeed linked to blackleg resistance in rapeseed. They provide an invaluable insight into genetically mediated rapeseed resistance to blackleg and have the potential to become a powerful tool in resistance breeding through MAS. Additionally, this research also confirmed that DArTseq-based GWAS is an efficient method for marker selection and mapping.

## Figures and Tables

**Table 1:** SilicoDArT and SNP molecular markers significantly associated with resistance of *B. napus* to blackleg at *p* < 0.001.

Marker	Marker Type	Chromosome	DNA Strand	Marker Position on Chromosome (bp)	Marker Sequence *
4899	SilicoDArT	A07	Minus	23,194,012–23,193,943	TGCAGGTTCTAAAAGAGTTTGAGTGGCGATCTGGTTTGGCAATGAGTGTGCAAAAAAGCTCCTTCTATG
5425	SilicoDArT	C03	Plus	1,036,696–1,036,759	TGCAGTCATGAAGGAACTTATCTTGAACTGCTTCAGTGCTTTGTATTCAATAATGAATTTTAC
5564	SilicoDArT	C02 random	Minus	4,895,158–4,895,089	TGCAGCTCACGTTGAGCTTCAAATTCTACGATCCAGATCCACACCACAATCTAGATCGATCATTTCCAA
5781	SilicoDArT	C02	Minus	44,607,251–44,607,182	TGCAGTAATTCCAACGTCCATCCTTCAGATCCAATGCCTTCATCTCAAGGTATTTTATTACTAATTTTG
6134	SilicoDArT	A07	Minus	14,719,379–14,719,339	TGCAGGAGAAGGGAAAATCAAAACTTCAATCCTTACTTAC
6776	SilicoDArT	A07	Plus	14,959,860–14,959,929	TGCAGCCACGACTGCAAATTTATCTGTTCGGATCAACGAGCCTAAGTCCAAACCTATTTTCTTGATTGG
7853	SilicoDArT	A06	Plus	5,968,268–5,968,337	TGCAGAAAATGGAATGTTCTTGAGAGATCCTAGTGGAGAATGGGTGACAAATATGCCTCAAGACATGAA
7889	SilicoDArT	C06	Plus	34,959,583–34,959,652	TGCAGAAGCAGCCATGAGACAGTATTGCTGTTGAGATATATTGTTGCTGTACCTTGGGGAGGAAGCAAC
9070	SilicoDArT	C06	Plus	21,498,041–21,498,110	TGCAGAATGCCAGGCTAAGTTGAGAGAAGAGAATCCAGGAAATGCACTCCTTGAGGTATATTCATATTC
10058	SNP (38:G>A)	A07	Plus	15,507,858–15,507,927	TGCAGTTGCACTTTCTCTTTCCAGGAGCTGGTTTTATAGCATTCTTCTCCCTCCATACCTGATAAATCA
11667	SNP (38:G>A)	A08	Minus	13,628,583–13,628,514	TGCAGCTTGTCAGCACATCCTCTCTTCACACACTCAATGATAAACACCTTGGCGTAACAATGAATCCGG
11720	SNP (18:A>T)	C06	Plus	34,959,583–34,959,652	TGCAGAAGCAGCCATGAGACAGTATTGCTGTTGAGATATATTGTTGCTGTACCTTGGGGAGGAAGCAAC
12134	SNP (29:G>A)	C06 random	Minus	2,170,846–2,170,777	TGCAGCTTCTACTTTTAGTTGGACAGAGCGCTCAAAGTCAACAATTACAGATCGGAAGAGCGGTTCAGC
12232	SNP (46:T>A)	C03	Plus	1,024,177–1,024,246	TGCAGAAAAAGATTCAGGTTCCCGGGACCTGAAGATCACTGGATTGTCTGATGCTGTGTTAGGATGCAT
12456	SNP (45:A>C)	A08	Plus	13,650,920–13,650,989	TGCAGTTTCTACACGTACATATCCAATATTTTAGTTTACTTAGGAAGAAATTTGAAATTTGATTTTATT

* SNPs within the marker sequences underlined.

**Table 2:** Candidate genes linked to the markers identified at *p* < 0.001.

Marker	Candidate Genes
4899	Sequence localized between *BnaA07g33940D* (5704 bp from START codon) and *BnaA07g33950D* (2160 bp from STOP codon)
5425	Sequence localized between *BnaC03g02170D* (2456 bp from STOP codon) and *BnaC03g02180D* (550 bp from START codon)
5564	Sequence localized between *BnaC02g48660D* (6400 bp from START codon) and *BnaC02g48670D* (3627 bp from START codon)
5781	Sequence localized within *BnaC02g41800D* (from 2nd exon to 2nd intron)
6134	Sequence localized within *BnaA07g17660D* (1st intron)
6776	Sequence localized within 16th (last) exon of *BnaA07g18190D*
7853	Sequence localized within 13th exon of *BnaA06g11460D*
7889	Sequence localized within 12th (last) exon of *BnaC06g36400D*
9070	Sequence localized within 4th exon and 4th intron of *BnaC06g19150D*
10058	SNP localized within 5th exon of *BnaA07g19340D*
11667	SNP localized within the only exon of *BnaA08g16950D*
11720	SNP localized within the 12th (last) exon of *BnaC06g36400D*
12134	SNP localized between *BnaC06g42650D* (1255 bp from START codon) and *BnaC06g42660D* (33,148 bp from STOP codon)
12232	SNP localized within the 9th exon of *BnaC03g02160D*
12456	SNP localized within the 1st intron of *BnaA08g17000D*

**Table 3:** Annotation of *Arabidopsis thaliana* orthologous genes. Data obtained from NCBI (https://www.ncbi.nlm.nih.gov/ (accessed on 15 March 2024)), BrassicaDB (http://www.brassicadb.org (accessed on 15 March 2024)) and TAIR (https://www.arabidopsis.org/ (accessed on 15 March 2024)) databases.

*B. napus* Gene	*A. thaliana* Orthologue	Protein Encoded by *A. thaliana* Orthologue	Protein Function
*BnaC02g41800D*	*At5g08130*	BES1-INTERACTING MYC-LIKE1, BIM1	Transcription factor Brassinosteroid signaling
*BnaA07g17660*	*At3g58180*	ARM repeat superfamily protein	Deoxyhypusine monooxygenase activity
*BnaA07g18190*	*At4g37950*	Rhamnogalacturonate lyase family protein	Enables carbohydrate binding, enables lyase activity, enables rhamnogalacturonan activity
*BnaA06g11460*	*At1g16980*	Trehalose-6-phosphate synthase	Enzyme putatively involved in trehalose biosynthesis
*BnaC06g36400D*	*At1g75730*	Hypothetical protein	---
*BnaC06g19150D*	*At1g80380*	*p*-loop containing nucleoside triphosphate hydrolases superfamily protein	Enables ATP binding, enables glycerate kinase activity, enables kinase activity
*BnaA07g19340*	*At3g62600*	DNAJ heat shock family protein	Enables Hsp70 protein binding, enables unfolded protein binding
*BnaA08g16950*	*At4g38690*	PLC-like phosphodiesterases superfamily protein	Enables phosphoric diester hydrolase activity
*BnaC03g02160D*	*At5g05560*	E3 ubiquitin ligase	Encodes a subunit of the *A. thaliana* E3 ubiquitin ligase complex that plays a synergistic role with APC4 both in female gametogenesis and in embryogenesis
*BnaA08g17000*	*At4g38600*	HECT ubiquitin protein ligase family protein KAK	Enables ubiquitin protein ligase activity, enables ubiquitin-protein transferase activity

## Data Availability

The data presented in this study are available on request from the corresponding author.
